# Keratin 79 is a PPARA target that is highly expressed by liver damage

**DOI:** 10.1016/j.bbrc.2023.01.071

**Published:** 2023-01-30

**Authors:** Donghwan Kim, Inwook Choi, Sang Keun Ha, Frank J. Gonzalez

**Affiliations:** a Division of Functional Food Research, Korea Food Research Institute, Wanju-gun, Republic of Korea; b Laboratory of Metabolism, Center for Cancer Research, National Cancer Institute, National Institutes of Health, Bethesda, MD, USA

**Keywords:** KRT79, Cytokeratin, PPARA, Liver

## Abstract

Keratins are key structural proteins found in skin and other epithelial tissues. Keratins also protect epithelial cells from damage or stress. Fifty-four human keratins were identified and classified into two families, type I and type II. Accumulating studies showed that keratin expression is highly tissue-specific and used as a diagnostic marker for human diseases. Notably, keratin 79 (KRT79) is type II cytokeratin that was identified as regulator of hair canal morphogenesis and regeneration in skin, but its role in liver remains unclear. KRT79 is undetectable in normal mouse but its expression is significantly increased by the PPARA agonist WY-14643 and fenofibrate, and completely abolished in *Ppara*-null mice. The *Krt79* gene has functional PPARA binding element between exon 1 and exon 2. Hepatic *Krt79* is regulated by HNF4A and HER2. Moreover, hepatic KRT79 is also significantly elevated by fasting- and high-fat diet-induced stress, and these increases are completely abolished in *Ppara*-null mice. These findings suggest that hepatic KRT79 is controlled by PPARA and is highly associated with liver damage. Thus, KRT79 may be considered as a diagnostic marker for human liver diseases.

## 1. Introduction

Keratins are the intermediate filament-forming protein in epithelial cells. The keratin family consists of 54 proteins in two families (type I, acidic, and II, neutral or basic), each being expressed in during differentiation [1]. Keratin proteins consist of simple, barrier and structural forms [2]. Keratins are normally expressed in a cell type-, differentiation- and functional status-dependent manner. Epithelial cancers largely maintain the characteristics of keratin expression associated with their respective cell type of origin [3]. Keratins have long been recognized as diagnostic markers in tumor pathology [3]. Notably, keratin 79 (KRT79) is type II keratins identified in a novel population of migratory epithelial cells that initiate hair canal morphogenesis and regeneration [4]. KRT79 has an important role in the maintenance of sebaceous gland stem cells in the skin [5]. Loss of KRT79 caused abnormal sebaceous glands and eyelid meibomian glands [5]. Proteomic analysis to identify the mechanism of the anticancer properties of flavonoids from *citrus platymamma* showed that KRT79 expression is a possible modulator of cell differentiation and cell motility, is down-regulated by *citrus platymamma* in A549 lung cancer cells [6]. A recent study showed that hepatic KRT23 is controlled by peroxisome proliferator-activated receptor α (PPARA) and the proto-oncogene MYC that are known to promote hepatocyte proliferation [7]. In the present study, basal expression levels of hepatic KRT79 was undetectable in mouse hepatocytes. but was highly induced by WY-14643 and fenofibrate, agonistic ligands for PPARA and completely abolished in *Ppara*-null mice. PPARA directly binds to the *Krt79* gene. Furthermore, PPARA-mediated liver stress by fasting and high-fat diet significantly induced hepatic KRT79 expression in mice and it was not found in *Ppara*-null mice. KRT79 is encoded by a PPARA target gene and is highly elevated in by liver damage.

## 2. Materials and methods

### 2.1. Cell culture

Hepa-1 cells (ATCC, Manassas, VA) were maintained at 37 °C in a humidified atmosphere of 5% CO_2_ in Dulbecco’s Modified Eagle Medium (DMEM) containing 10% Fetal Bovine Serum (FBS) and 1% of penicillin/streptomycin mixture (Invitrogen, Waltham, MA).

### 2.2. Primary hepatocyte isolation

previously reported [8] and seeded on collagen-coated 12-well plates (Becton Dickinson and Company) at a density of 2 × 10^5^ cells in Williams’ Medium E (Thermo-Fisher Scientific, Waltham, MA) supplemented with 5% FBS and penicillin/streptomycin/amphotericin B solution (Gemini Bio-products). The cells were harvested and subjected to qRT-PCR for mRNA quantification.

### 2.3. Quantitative reverse transcription PCR (qRT-PCR) assays

Total RNA was isolated from fresh mouse liver, mouse primary hepatocytes, and Hepa-1 cells using TRIzol Reagent (Thermo-Fisher Scientific) and quantified using a NanoDrop Spectrophotometer (NanoDrop Products, Wilmington, DE). Total RNA (2 mg) was reverse transcribed using All-in-One cDNA Synthesis SuperMix (BioTool, Houston, TX). qRT-PCR analysis was performed using SYBR Green qPCR Master Mix (BioTool). The primers sequence were as follows, *Krt79*: forward 5′-GCAATCAGCATCTCGGTGAC-3′, reverse 5′-CAGCGCCATAGCTCACACTA-3’; *Krt79* exon 1: forward 5′-TGACTATGTCCAGGGGGAGC-3′, reverse 5′-CAAGCCATGCTGACGGAGAT-3’; *Krt79* exon 2: 5′-AGCCGGAGCCTCTACAACTT-3′, reverse 5′-ACTGCCAAAGCCTCCTAGTG-3’; *Krt79* exon 4: forward 5′-TGTAGATGCCGCATACATGG-3′, reverse 5′-CAAAGAGCTGTTGCAGGAAGTC-3’; Gapdh: forward 5′-GACTTCAACAGCAACTCCCAC-3′, reverse 5′-TCCACCACCCTGTTGCTGTA-3’. Primers were designed for mRNA specificity and to cross exon-exon junctions using Primer-BLAST (www.ncbi.nlm.nih.gov/tools/primer-blast/) and purchased from IDT DNA Technologies (Coralville, IA). qRT-PCR experiments were designed and performed according to Minimum Information for Publication of Quantitative Real-Time PCR Experiments (MIQE) guidelines [9]. Results are normalized to actin expression. Values given are fold over control or relative expression value, where appropriate, calculated using the 2DCt QPCR calculation method [10].

### 2.4. Immunohistochemistry of KRT79

Immunohistochemical staining was performed on formalin-fixed paraffin embedded sections from human or mouse liver. For immunohistochemical staining, sections were deparaffinized followed by antigen retrieval with sodium citrate buffer for 2 min at 95 °C and then placed at room temperature (RT) for 30 min. Sections were blocked using 5% BSA for 30 min followed by incubation with 10% goat serum for 30 min at RT. After blocking, sections were incubated with KRT79 (Thermo-Fisher Scientific; PA5–46517) primary antibody in a humidified chamber overnight at 4 °C. Following primary antibody incubation, sections were washed and incubated with secondary antibodies for 30 min (Gene Tech Company; GK600505). Following secondary antibody incubation, the sections were washed, and DAB substrate added (Vector Laboratories, Burlingame, CA) and developed by checking for staining under a microscope. The sections were then washed and counterstained with hematoxylin for 30 s. Sections were then washed with tap water, dehydrated, and mounted. All imaging was performed using a Keyence BZ-X700 series all-in-one microscope with 20x and 40x objectives (200x magnifications) (Keyence, Osaka, Japan).

### 2.5. Luciferase reporter assays

For luciferase assays, pSG5-PPARA (mouse) and pSG5-RXRA (mouse) were used for transcription factor expression [11]. Custom GeneBlocks (IDT DNA, Coralville, IA) were synthesized containing the predicted PPRE sites on *Krt79* gene. GeneBlocks were digested and purified using a Qiagen PCR Purification Kit (Qiagen, Valencia, CA), and cloned into the pGL4.11 for PPRE constructs (Promega, Madison, WI) using a BioRad Quick Ligation Kit (BioRad, Hercules, CA). Prior to performing assays, all constructs were confirmed by Sanger sequencing at the NCI Center for Cancer Research Genomics Core. The phRL-TK renilla luciferase construct was used as a control to normalize for transfection efficiency. Primary hepatocytes were seeded into 12-well plates (4 × 10^4^ cells/well). PPRE reporter constructs were co-transfected into hepatocytes with PPARA and RXR expression. vectors. PPRE-luc plasmid containing an *Acox1* peroxisome proliferator regulatory element (PPRE) site was used as a positive control [12]. Empty pGL4.11 plasmid was used as negative controls. Plasmids were transfected using Lipofectamine 3000 Reagent (Thermo-Fisher Scientific). Luciferase activities were measured and plotted relative to lysate protein concentrations using the Promega Dual Luciferase Reporter (DLR, Promega) assays according to the manufacturer’s protocol. Measurements were taken on a Veritas microplate luminometer (Turner Biosystems, Sunnyvale, CA).

### 2.6. Mouse models

*Ppara* wild-type (*Ppara*^−/−^) male mice and conventional *Ppara*^−/−^ male mice used in this study were described [13]. For WY-14643 studies, mice were provided a grain-based control diet or matched diet containing 0.1% WY-14643 for 2 days. For monitoring the time dependence of *Krt79* gene responses, WY-14643 was dissolved in 1% carboxymethyl cellulose (CMC) solution and orally administered (50 mg/kg in 200 μl) for the indicated time points. Hepatocyte nuclear factor 4α (*Hnf4a*) wild-type male mice and hepatocyte-specific *Hnf4a*-null (H*nf4a*^Δ*He*p^) male mice used in this study were described [14]. For estrogen receptor activation studies, 2 mg of tamoxifen was intraperitoneally injected. For fasting studies, food was removed for 24 h starting shortly after the onset of the light cycle and endpoints collected at the same time the following day. For the HFD feeding experiment, mice were fed a HFD (60% kcal from fat) for 16 weeks. Mice were housed in light and temperature–controlled rooms and were provided with water and pelleted chow ad libitum. Mice were then killed, and tissue samples harvested, snap frozen in liquid nigrogen and stored at −80 °C for further analysis. All animal experiments were performed in accordance with the Association for Assessment and Accreditation of Laboratory Animal Care international guidelines and approved by the National Cancer Institute Animal Care and Use Committee.

### 2.7. Computational and statistical analysis

All results are expressed as means ± SD. Significance was determined by *t*-test or one-way ANOVA with Bonferroni correction using Prism 7.0 software (GraphPad Software, La Jolla, CA). A P value less than 0.05 was considered significant and statistical significance is indicated in the figure legends.

## 3. Results

### 3.1. KRT79 response is PPARA-dependent and liver specific

**Fig. 1.**
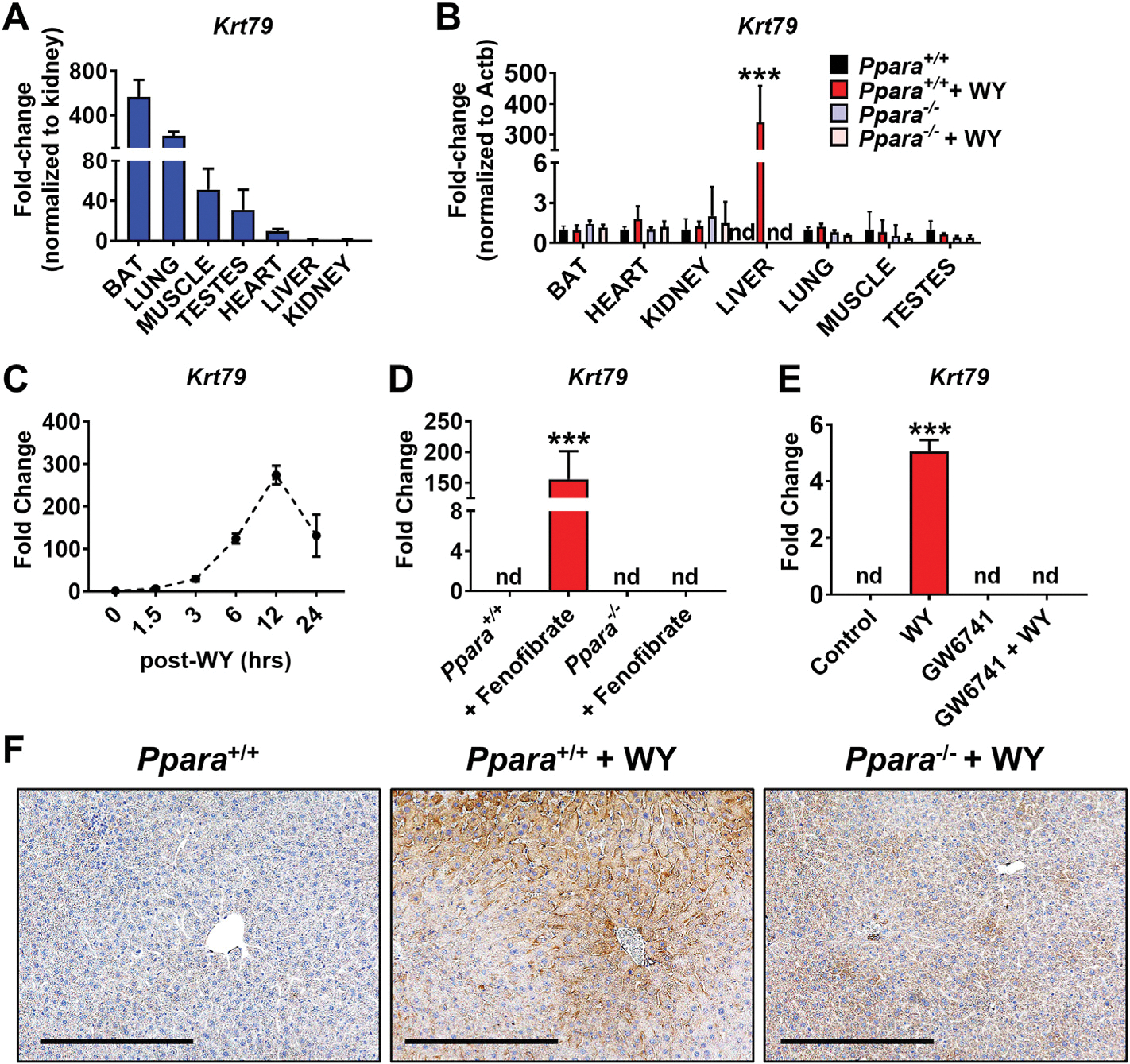
Liver-specific KRT79 responds to PPARA. *Ppara*^+/+^ and *Ppara*^−/−^ mice were treated with WY-14643 for 48 h. (**A**) qRT-PCR analysis of *Krt79* in various tissues. (**B**) Expression of *Krt79* mRNA in *Ppara*^+/+^ and Ppara^−/−^ mice with and without WY-14643. (**C**) Time course for changes in expression of *Krt79* mRNA over a 24 h period following treatment with WY-14643 by gavage, determined by qRT-PCR. The maximum response of *Krt79* mRNA was seen at 12 h. (**D**) Expression of *Krt79* mRNA in *Ppara*^+/+^ and *Ppara*^−/−^ mice with and without fenofibrate. (**E**) Expression of Krt79 mRNA in primary hepatocyte from mice treated with WY-14643 (50 μM) or treated with GW6741(5 μM) and treated with WY-14643 + GW6741 for 24 h. (**F**) IHC staining of KRT79 in liver sections from control mice, WY-14643-treated wild-type and WY-14643-treated *Ppara*^−/−^ mice. Representative images are shown. Scale bars represent 100 nm (200 ×). Each data point represents the mean ± SD for n = 5 liver samples. ***P < 0.001. nd, no detection.

**Fig. 2.**
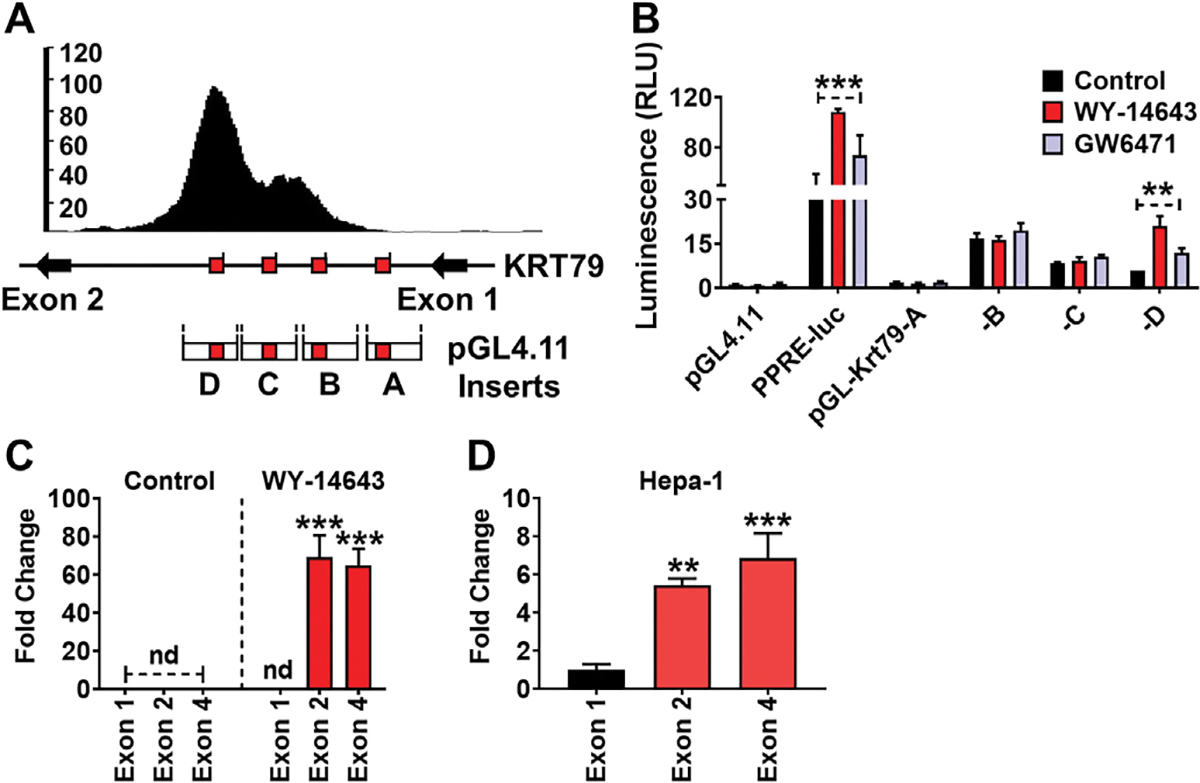
PPARA directly regulates *Krt79*. **(A)** Schematic representation of predicted PPRE sites found within the *Krt79* gene. Reporter construct inserts are shown below. **(B)** Luciferase-based reporter assays confirmed functional PPREs sites found between exon1 and exon 2 of *Krt79*. **(C)** qRT-PCR analysis of *Krt79* exon 1, 2, and 4 in liver tissue. **(D)** qRT-PCR analysis of *Krt79* exon 1, exon 2, and exon 4 in Hepa-1 cells. Experiments were performed with at least four replicates. Each data point represents the mean ± SD. **P < 0.01; ***P < 0.001. nd, no detection.

Basal expression levels of *Krt79* mRNA were highest in brown adipose, lung and muscle but very low in liver (Fig. 1A). KRT23 is type I cytokeratin also highly induced by ligand activation of PPARA [7]. Induction of *Krt79* mRNA by WY-14643 was liver specific and not observed in *Ppara*^−/−^ mice, indicating that their expression is PPARA dependent (Fig. 1B). *Krt79* mRNA was rapidly induced with maximum expression seen at 12 h after ligand administration (Fig. 1C). Fenofibrate treatment increased *Krt79* mRNA expression and not observed in *Ppara*^−/−^ mice (Fig. 1D). In primary hepatocytes, *Krt79* mRNA was not increased by the PPARA antagonist GW6741 which also block its induction by WY-14632 (Fig. 1E). KRT79 staining was primarily cytosolic and was increased significantly in response to WY-14643 treatment when compared with controls; staining was markedly decreased in *Ppara*^−/−^ mice (Fig. 2F). These data show that *Krt79* mRNA expression in response to PPARA activation was liver specific, indicating a potential role for these proteins in hepatocyte proliferation.

### 3.2. PPARA directly regulates KRT79 by binding to promoter response elements

Genomatix MatInspector (Genomatix, Munchen, Germany) was used to bioinformatically identify potential PPREs on the Krt79 gene. Four PPRE sites were identified in the intron between *Krt79* exon 1 and 2 (Fig. 2A). Inserts (A-D) containing the predicted PPRE sites were synthesized and cloned into the pGL4.11 reporter and luciferase assays performed to confirm direct PPARA binding on the *Krt79* gene. Another PPRE-luc construct containing an *Acox1* PPRE repeat was used as a positive control and empty pGL4.11 plasmid was used as a negative control. Luciferase activity was significantly elevated in primary hepatocytes transfected with the pGL4.11-D construct which contained a single PPRE site, indicating direct regulation by PPARA (Fig. 2B). To check whether exon 1 is amplified after PPARA binding, exon specific primers were designed and subjected to mRNA quantification. *Krt79* exon 1 is not transcribed in mouse liver after WY-14643 treatment (Fig. 2C). In Hepa-1 cells, *Krt79* exon 1 RNA transcript level was very low compared to exon 2 and exon 4 RNA transcripts (Fig. 2D). Together these data indicate that activation of PPARA regulates *Krt79* transcription primarily through direct binding to a PPRE sites found in the intron between exons 1 and 2.

### 3.3. KRT79 expression is affected by hepatocyte nuclear factor 4α and estrogen receptor

**Fig. 3.**
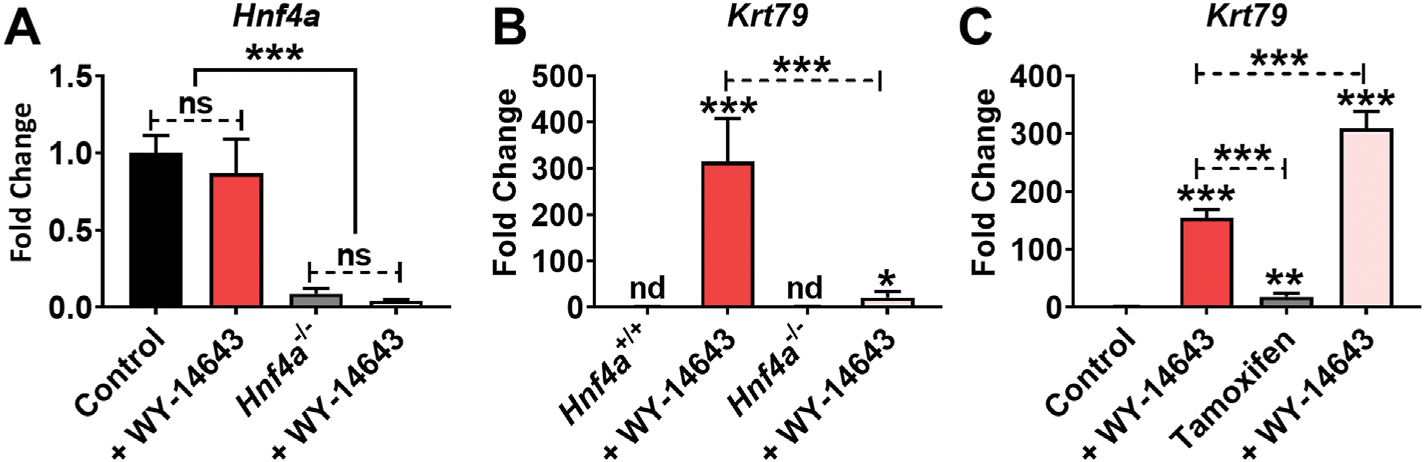
Hepatic KRT79 is regulated by HNF4A and ER. (A) qRT-PCR analysis of *Hnf4a* in *Hnf4a*^+/+^ and *Hnf4a*^ΔHep^ mice with and without WY-14643. (B) qRT-PCR analysis of *Krt79* mRNA in *Hnf4a*^+/+^ and *Hnf4a*^ΔHep^ mice treated with and without WY-14643. (C) qRT-PCR analysis of *Krt79* mRNA in WY-14643-treated mice treated with and without 2 mg of tamoxifen. Each data point represents the mean ± SD for n = 5 liver samples. *P < 0.05; **P < 0.01; ***P < 0.001. ns, no significant. nd, no detection.

Bioinformatical analysis by Genomatix MatInspector (Genomatix, Munchen, Germany) revealed that the *Krt79* gene has hepatocyte nuclear factor 4 α (HNF4A) and estrogen receptor (ER) binding elements. To identify whether hepatic Krt79 expression is affected by HNF4A and ER, *Hnf4a*^ΔHep^ mice were treated WY-14643, and the ER antagonist tamoxifen was administered to wild-type mice. Hepatic *Hnf4a* mRNA expression was decreased in *Hnf4a*^ΔHep^ mice treated with and without WY-14643 (Fig. 3A). However, WY-14643-induced hepatic *Krt79* mRNA expression was significantly inhibited in H*nf4a*^ΔHep^ mice treated with WY-14643 (Fig. 3B). Tamoxifen is breast cancer drug that competitively inhibits activation of the ER [15]. Tamoxifen treatment induced hepatic *Krt79* expression (Fig. 3C). Interestingly, hepatic *Krt79* expression was significantly increased in combination with WY-14643 and tamoxifen (Fig. 3C). These data suggest that hepatic *Krt79* expression is regulated by HNF4A and ER (Fig. 3).

### 3.4. KRT79 expression is highly elevated in liver damage

**Fig. 4.**
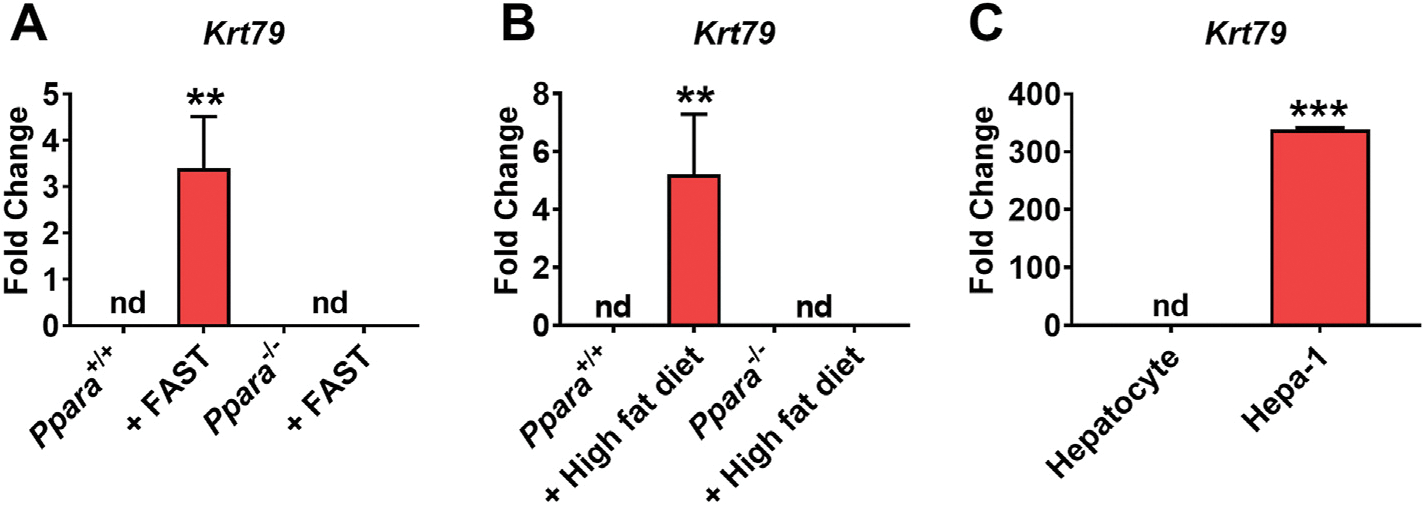
*Krt79* expression is elevated in liver damage. **(A)** qRT-PCR analysis of *Krt79* in *Ppara*^+/+^ and *Ppara*^−/−^ mice treated with and without 24 h fasting in liver. **(B)** qRT-PCR analysis of *Krt79* in *Ppara*^+/+^ and *Ppara*^−/−^ mice with and without high fat diet in liver. **(C)** qRT-PCR analysis of *Krt79* mRNA in primary hepatocyte and Hepa-1 cells. Each data point represents the mean ± SD for n = 5 liver samples. **P < 0.01; ***P < 0.001. nd, no detection.

Recent studies showed that keratins are histological cancer diagnostic markers, particularly epithelial cell-derived cancers [16,17]. KRT23 is PPARA dependent and also highly expressed in human liver cancer [7]. To determine whether KRT79 is associated with liver disease, fasting and high-fat diet-induced liver disease models were analyzed for *Krt79* expression. *Krt79* mRNA expression was significantly increased in liver of fasted and high-fat diet treated mice; it was not elevated in liver of *Ppara*^−/−^ mice under these treatments (Fig. 4A–B). Basal expression levels of *Krt79* mRNA is highly elevated in Hepa-1 cells compared to primary hepatocyte (Fig. 4C). These data indicated that KRT79 is highly associated with liver damage.

## 4. Discussion

Keratins are expressed in all type of epithelial tissues and play an important role in epithelial cell protection from stressors [18]. Keratins are conventionally thought of as structural proteins, but more recently, several studies have revealed that they also play active roles in cell signaling pathways [19,20]. This is first study to investigate the role of KRT79 in liver. *Krt79* mRNA was abundant in brown adipose, lung, and muscle tissues. Basal expression of hepatic *Krt79* mRNA was undetectable in livers of untreated wild-type mice. However, the PPARA agonist WY-14643 significantly induced *Krt79* mRNA and KRT79 positive cells in livers of wild-type mice. *Krt79* mRNA was unchanged in *Ppara*^−/−^ mice treated with a PPARA agonist, indicating that *Krt79* induction is PPARA dependent. *Krt79* is robustly increased beginning 3 h after activation of PPARA by WY-14643. Hepatic *Krt79* mRNA was also induced by fenofibrate in mice and by WY-14643 in primary hepatocyte cultures. These data revealed that hepatic *Krt79* is PPARA target gene.

PPARA ChIP-seq analysis revealed that the *Krt79* gene has potential PPARA binding sites (GSE61817). The underlying PPARA regulatory interactions controlling activation of the *Krt79* promoter were investigated. PPARA binding sites were functionally identified in the intron between exons 1 and 2 of the *Krt79* gene as revealed by luciferase reporter gene assays. To determine whether the expression of exon 1 RNA is increased after PPARA binding, qRT-PCR was perfomed on exons 1, 2, and 4 derived RNAs. The results showed that *Krt79* exon 1 was not transcribed in WY-14643 treated mouse liver. Krt79 exon 1 transcripts were significantly lower than exon 2 and 4 transcripts in Hepa-1 cells. This result suggest that *Krt79* exon 1 may act as negative regulator of *Krt79*. Thus, hepatic *Krt79* is a direct PPARA target gene in WY-14643-stimulated mouse liver.

Bioinformatical analysis revealed that the *Krt79* gene has multiple transcription factor binding elements including those for HNF4A and ER. WY-14643-induced hepatic *Krt79* was significantly inhibited in *Hnf4a*^ΔHep^ mice treated with WY-14643. Tamoxifen is an anticancer and chemoprevention drug for ER-positive breast cancer, acting as an ER antagonist [15,21]. Surprisingly hepatic *Krt79* mRNA was also increased by tamoxifen. Notably, treatment with WY-14643 and tamoxifen caused significantly increased hepatic *Krt79* mRNA expression compared to treatment with WY-14643 or tamoxifen alone. These data suggested that HNF4α and ER can regulate hepatic *Krt79* when *Krt79* is activated by PPARA.

Fasting and high fat diet-induced pathological liver stress result in PPARA activation by endogenous ligands [22,23]. Fasting and high fat diet also induced hepatic *Krt79* mRNA expression, that was not found in liver of *Ppara*^−/−^ mice. Furthermore, basal expression level of *Krt79* in Hepa-1 cells originated mouse hepatoma was significantly elevated compared to primary hepatocytes. These data indicated that hepatic KRT79 is highly associated with liver damage. Taken together, these results demonstrate that hepatic Krt79 is a PPARA target gene and that *KRT79* can be used as a biochemical or histological diagnostic biomarker for human liver diseases.
